# Quality of life in different geographic areas; sharing an experience

**Published:** 2013-07-01

**Authors:** Behzad Einollahi, Mohsen Motalebi

**Affiliations:** ^1^Nephrology and Urology Research Center, Baqiyatallah University of Medical Sciences, Tehran, Iran

**Keywords:** Quality of life, Dialysis, Kidney disease

**Implication for health policy/practice/research/medical education:**

Various factors such as perception of social support, expectations of life, healthy behaviors and outlooks, religious conviction may affect quality of life in dialysis patients.

Recently much attention had been directed toward the quality of life in dialysis patients. The published article by Cavalcante *et al.*, titled “factors associated with the quality of life of adults subjected to hemodialysis in a city in northeast Brazil”(1), has focused on exploration of socioeconomic, demographic, clinical and laboratory factors that can affect quality of life (QoL) in hemodialysis (HD) patients in Brazil. We have also conducted a study to evaluate QoL on 6930 HD patients in Iran (2) applying the kidney disease component summary - Short Form 1.3 questionnaire (KDCS-SF). We would like to share our experience that may be helpful to others. Cavalcante *et al*. (1) found employment status, burden of kidney disease, general health, patient satisfaction and physical function were the domains with worsen QoL (≤50). We have also shown work status, burden of kidney disease, general health and physical function were the domains with worsen QoL (2) as well as role physical, role emotional and energy\fatigue as shown in Table 1, of course with lower scores compared to current study (2). The low scores of physical domains in both studies obviously show that spending many times for hemodialysis can affect and disturb patient’s daily activities every week. In addition, majority of QoL domains scores among our HD patients were lower than patients on Cavalcante *et al*. study, which indicated the difference of perception of QoL between two countries’ people. Kutner *et al*. showed there are differences between QoL between white and black races (3). So it seems many factors such as perception of social support, expectations of life, healthy behaviors and outlooks, religious conviction and etc. are different among countries. It is interest of that Cavalcante *et al*. showed gender had no effect on HD patients’ QoL, while we showed better QoL in males than that of females. Zender *et al.* (4) also reported a lower QoL in women when compared to men; in addition, they showed a high prevalence of psychological disorders with more severity that it can lead to lower QoL in females. We think it is due to differences of male roles in family and community between both countries. Although the current study shows dialysis duration has no effect on patients QoL, we found that there was the significant correlation between QoL and dialysis duration (2) so that more dialysis duration patients had lower QoL. Bayoumi *et al*. also considered that dialysis duration was a negative predictor for QoL (5). This different outcome may be due to two reasons: 1) patients older than 60 years old were 39.7% in our study while all of patients in the present study (1) were younger than this. 2) patients size in the present study (1) was smaller compared to our study which had a huge number of patients. Finally, we suggest a multi-center study or a meta-analysis to evaluate QoL in different countries to determine whether different geographic areas have effect on QoL or not.

**Table 1:** Mean and Standard Deviation, of KDQOL-SF and SF-36 Items (Ref. 2)

	**Mean ± SD**
**Symptoms**	67.9 ± 19.8
Effects of kidney disease	50.42 ± 20.9
Burden of kidney disease	23.08 ± 19.78
Work status	22.3 ± 34.56
Cognitive function	66.26 ± 21.21
Quality of social interaction	67.07 ± 20.08
Sexual function	63.48 ± 30.4
Sleep	55.9 ± 19.9
Social support	72.8 ± 26.9
Dialysis staff encouragement	81.3 ± 21.87
Patient satisfaction	69.01 ± 24.24
Kidney disease component summary (KDCS)	57.97 ± 11.7
Physical function	40.46 ± 29.5
Rolephysical	25.6 ± 32.7
Pain	55.31 ± 25.73
General health	41.70 ± 19.71
Physical component summary (PCS)	40.79 ± 20.1
Emotional well-being	54.24 ± 18.03
Role emotional	36.28 ± 38.89
Social function	55.77 ± 22.3
Energy/ fatigue	44.76 ± 19.79
Mental component summary (MCS)	47.79 ± 18.31
SF-36	44.29 ± 17.7
SF-36 + KDCS	51.12 ± 13.41

## Authors’ contributions

All authors contributed to study equally.

## Ethical considerations

Ethical issues (including plagiarism, informed consent, misconduct, double publication and redundancy) have been completely observed by authors.

## Conflict of interests

The authors declared no competing interests.

## Funding/Support

No funding from any source.
